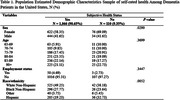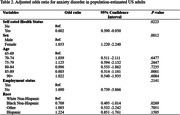# Association between Self‐rated Health Status and Anxiety Symptoms in U.S. Adults with Dementia

**DOI:** 10.1002/alz.088660

**Published:** 2025-01-09

**Authors:** Ickpyo Hong, HOKYUNG LEE

**Affiliations:** ^1^ Yonsei University, Wonju, Gangwon‐do Korea, Republic of (South)

## Abstract

**Background:**

Self‐rated health status goes beyond a mere indicator of one’s well‐being, encompassing biological, social, and functional aspects. It has emerged as a significant variable in predicting mortality. Against this backdrop, understanding the impact of subjective health status on anxiety symptoms is crucial for individuals with dementia. In patients with dementia, subjective health status reflects personal perception. Understanding how this relates to their anxiety symptoms can help develop effective health management and support strategies for them.

**Method:**

This study utilizes the 2022 National Health and Aging Trends Study (NHATS). The relationship between subjective health status and anxiety symptoms among American adults diagnosed with dementia was examined using multivariate logistic regression analysis. The independent variable in this study was the self‐reported subjective health status, either positive or negative, among patients with dementia. The dependent variable was the level of anxiety symptoms, measured using the GAD‐2 questionnaire. Covariates include sex, age, race, and employment status.

**Result:**

In this study of 1,176 subjects, a majority (90.65%) reported a positive perception of their health status. Demographically, women constituted a slightly larger proportion (58.35%), and the positive evaluation of subjective health status was predominantly observed in those aged over 80. The findings reveal a significant difference between dementia patients who perceived their health status positively and those who did not.. In particular, those with positive subjective health status experienced less anxiety, and women reported more anxiety symptoms than men (P = .0223, OR = 0.602; P < .05, OR = 1.653). Age and race differences in anxiety symptoms were generally not statistically significant, while black individuals tended to experience less anxiety than white individuals (P = .0269, OR = 0.709).

**Conclusion:**

This study indicated a significant association between subjective health status and anxiety symptoms among adult dementia patients in the United States. The majority of participants positively assessed their health, and this positive subjective health perception was associated with a reduction in anxiety symptoms. These findings underscore the importance of personalized approaches in mental health management for dementia patients, taking into account gender and racial factors.